# Ethical climate and moral distress in paediatric oncology nursing

**DOI:** 10.1177/0969733021994169

**Published:** 2021-03-11

**Authors:** Päivi Ventovaara, Margareta af Sandeberg, Janne Räsänen, Pernilla Pergert

**Affiliations:** 27106 Karolinska Institutet, Sweden; Karolinska University Hospital, Sweden; 60670 Tampere University Hospital, Finland; 27106 Karolinska Institutet, Sweden; Karolinska University Hospital, Sweden

**Keywords:** Ethical climate, moral distress, nurses, paediatric oncology

## Abstract

**Background:**

Ethical climate and moral distress have been shown to affect nurses’ ethical behaviour. Despite the many ethical issues in paediatric oncology nursing, research is still lacking in the field.

**Research aim:**

To investigate paediatric oncology nurses’ perceptions of ethical climate and moral distress.

**Research design:**

In this cross-sectional study, data were collected using Finnish translations of the Swedish Hospital Ethical Climate Survey–Shortened and the Swedish Moral Distress Scale–Revised. Data analysis includes descriptive statistics and non-parametric analyses.

**Respondents and research context:**

Ninety-three nurses, working at paediatric oncology centres in Finland, completed the survey.

**Ethical considerations:**

According to Finnish legislation, no ethical review was needed for this type of questionnaire study. Formal research approvals were obtained from all five hospitals. Return of the questionnaire was interpreted as consent to participate.

**Results:**

Ethical climate was perceived as positive. Although morally distressing situations were assessed as highly disturbing, in general they occurred quite rarely. The situations that did appear often reflected performing procedures on school-aged children who resist such treatment, inadequate staffing and lack of time. Perceptions of ethical climate and frequencies of morally distressing situations were inversely correlated.

**Discussion:**

Although the results echo the recurrent testimonies of busy work shifts, nurses could most often practise nursing the way they perceived as right. One possible explanation could be the competent and supportive co-workers, as peer support has been described as helpful in mitigating moral distress.

**Conclusion:**

Nurturing good collegial relationships and developing manageable workloads could reduce moral distress among nurses.

## Introduction

Caring for sick children with life-threatening illnesses, such as cancer, exposes nurses to numerous ethical challenges. Despite good intentions to provide ethically good care, personal and professional moral values are challenged in clinical practice. This becomes apparent when paediatric nurses perform uncomfortable medical procedures that cause pain to children or when protective parents limit truth-telling.^1^ Furthermore, conflicting perspectives on medical decisions may appear between parents, nurses and other healthcare professionals, which can easily cause disagreements about what is in the best interest of the child.^1^ Nurses’ responses to ethical challenges are influenced by the ethical climate.^2^

Ethical climate has attracted much attention due to its impact on nurses and on patient care. The interest in ethical climate was sparked by Victor and Cullen who described it as ‘the shared perceptions of what is ethically correct behaviour and how ethical issues should be handled’.^3^ The concept was brought into healthcare by Olson, who developed an instrument to measure nurses’ perceptions of ethical climate.^4^ According to a Swedish study, less than one-third of the paediatric nurses had a positive perception of their possibilities to practise ethically good care.^5^ Other studies have suggested an association between ethical climate and other work-related factors such as job satisfaction and moral distress.^6^

When dealing with things beyond their own control and not being able to do the right thing for their patients, nurses encounter discomfort and anguish. These feelings have been comprehended as moral distress since the 1980s when Jameton coined the term.^7^ So far, moral distress in paediatric healthcare has been mostly studied among nurses working in paediatric and neonatal intensive care units. Nurses have identified issues anchored in end-of-life care and futile treatments as prominent causes of moral distress, together with unsafe staffing levels.^8,9^ The few studies carried out exclusively in paediatric oncology care have suggested that incompetence of healthcare providers and poor communication are other common challenging issues.^10,11^ Unattended, moral distress can lead to burnout, anger, sadness, frustration, intention to leave a clinical position, withdrawal from patients and diminished quality of patient care.^12,13^

Prior research has found ethical climate and moral distress to be linked with each other.^6^ This association has not yet been fully explored in paediatric oncology nursing, but mostly in adult care settings.^6^ To fill the gap in current research, a multi-centre project was initiated to investigate ethical climate and moral distress in paediatric oncology care in Nordic countries. To study conditions and practices that influence nurses’ ethical behaviour can result in implications for clinical practice and further research.

## Research aim and research questions

In this study, as a part of the multi-centre project in the Nordic countries,^10,14,15^ we aimed to explore nurses’ experiences of the ethical climate and moral distress in Finnish paediatric oncology care. Our research questions were as follows:

- What are the nurses’ perceptions of the ethical climate at their workplace?
- What are the nurses’ perceptions of moral distress in different situations?
- Do perceptions of moral distress differ between nurses according to work experience in paediatric healthcare and to level of education?
- Are perceptions of the ethical climate correlated with levels of moral distress?

## Research design

This descriptive, cross-sectional study was carried out in the framework of the Nordic Society for Paediatric Haematology and Oncology (NOPHO) and the Nordic Society of Paediatric Oncology Nurses (NOBOS).

### Respondents and research context

We investigated nurses’ perceptions of the ethical climate and moral distress in Finnish paediatric oncology care. The study was conducted at the five paediatric oncology centres located at five University Hospitals in Finland.

The study was carried out in collaboration with the centres. After an initial contact with the head nurses, research permission was obtained from all of the hospitals. Each centre decided upon a suitable starting point. A local coordinator informed personnel about the study and performed the data collection. Altogether, 169 registered nurses, 32 physicians and 9 nursing assistants (n = 210) were invited to complete a pen-and-paper questionnaire. However, this article reports exclusively on registered nurses’ perceptions (n = 169). The five data collections were carried out between May 2019 and January 2020, and the collection periods varied between centres from 3 weeks to 3 months.

### Instruments

The questionnaire included questions about the respondents’ demographic characteristics and the survey instruments.

*The Swedish Hospital Ethical Climate Survey–Shortened (Swedish HECS-S)* was translated into Finnish as described below. The original HECS was developed in the 1990s by Olson,^4^ who organised the statements according to nurses’ interactions with other nurses (peers), physicians, patients, managers and the hospital. Later on, the instrument was shortened, and parallel versions were created for nurses, physicians and other professionals.^16,17^ The second and the last author of this study coordinated the process of translating and adapting the Swedish HECS-S,^18^ with the permission of Dr AB Hamric (email, 15 November 2014) and Dr L Olson (email, 29 May 2015). The Swedish HECS-S is a multi-professional tool intended to measure perceptions of nurses, physicians and nursing assistants, and therefore, the original term ‘peers’ targeting other nurses has been renamed ‘co-workers’ to cover all professions.^18^ Consequently, the Swedish HECS-S assesses a dimension of relationships between co-workers rather than relationships to peers. All items are rated on a 5-point scale with labelled end points, ranging from 1 (*hardly ever*) to 5 (*almost always*). The higher scores imply more positive perceptions. While the Swedish HECS-S consists of 21 items, it was reduced to 18 items in the present analysis, due to irrelevance of three questions aimed at nursing assistants, as in two participating centres nursing staff consisted of registered nurses only.

*The Swedish Moral Distress Scale–Revised (Swedish MDS-R)* was translated into Finnish as described below. The original MDS was developed by Corley et al. to investigate moral distress among intensive care nurses.^19^ It has since been shortened^16^ and revised for other professions and settings.^20^ The instrument has earned an extreme popularity in various healthcare settings and cultures. The items describe clinical situations and internal and external constraints^20^ that healthcare providers may experience as disturbing: end-of-life care and quality of life, poor communication, staffing and material resources, hierarchies of decision-making and witnessing unethical behaviour.^21^ The second and the last author of this study coordinated the process of translating and adapting the Swedish MDS-R et al,^15,22^ with permission from Dr AB Hamric (email, 6 October 2014), for multi-professional use in paediatric oncology care. The Swedish MDS-R consists of 26 items whereof each is rated twice: first, how disturbing (intensity) the situation is assumed to be, and second, how often (frequency) the situation occurs. Items are scored on a 5-point scale with labelled end points: the intensity scores range from 0 (*not at all*) to 4 (*very negatively*), and the frequency scores range from 0 (*never*) to 4 (*very often*). The higher scores indicate higher moral distress levels.

*Translation process and validity.* Content validity of the Swedish versions of the HECS-S and the MDS-R had been assessed in preceding Swedish studies.^10,14,15^ The Swedish HECS-S and the Swedish MDS-R were translated from Swedish to Finnish using a translation method described by Daniel et al.^23^ with an aim to attain conceptual and content equivalence. The process involved two translations, one by the first author, who is a native Finnish speaker and fluent in Swedish, and another by a professional translator. These two translations were compared and converged during a group interview with three native Finnish speaking healthcare professionals working at a paediatric oncology centre in Sweden. Thereafter, face validity of the Finnish questionnaire was tested in six cognitive interviews with native Finnish-speaking healthcare professionals, using the Think Aloud technique.^24^ Continual discussions were held between the authors, and some changes were made after comparing, when applicable, wording with an existing Finnish version of the original HECS,^25^ after obtaining permission (email, R Suhonen, 19 December 2018). Translating the MDS-R from Swedish (a Germanic language) to Finnish (a Uralic language) provided linguistic challenges while attempting to maintain similar sentence structure.

### Analysis

Data were analysed using IBM SPSS Statistics 25. Questionnaires with less than 10% missing answers were included in the analysis. To judge the reliability of the instruments, Cronbach’s alpha values were calculated. Frequencies and central tendencies were calculated to summarise the data. Categorical data were described as counts and percentages and ordinal data as means with standard deviations (SDs). The decision to present means was based on current praxis in the field, to enable comparisons between studies. Mean values per respondent were calculated for each scale, and the differences between groups were analysed with Mann–Whitney *U*-test. A Spearman’s rank-order correlation was run to evaluate the relationships between mean values. In all analyses, *p* < 0.05 was considered as statistically significant.

## Ethical considerations

The study followed the ethical principles for conducting research.^26^ The Finnish National Board of Research Integrity (email, P Louhiala, 28 February 2018) informed us of the Finnish legislation that does not require ethical review for questionnaire studies. Applications for research permissions were made to each hospital. All five University Hospitals reviewed and approved the study (50H116, 62/2019, 110/2019, HUS/284/2019, T08/014/19). Research applications included inter alia a description of data protection required by the EU General Data Protection Regulation (GDPR). A cover letter was attached to the questionnaires, explaining the purpose of the study and the voluntary nature of participation. Respondents were assured that all data would remain confidential. Formal signed consents were not obtained; instead, the returning of the questionnaire was interpreted as consent to participate.

## Results

Altogether, 169 nurses who were on duty during the data collection periods received the questionnaire, and 96 (57 %) of them returned it. The response rate varied between centres from 32% to 83%. Three nurses were excluded from this study as they did not provide direct patient care, and thus, this study includes 93 nurses. However, one respondent had bypassed the HECS-S instrument and six other respondents had left more than 10% of the MDS-R questions unanswered, motivating their exclusion from the respective analysis. Thus, the total number of valid responses was 92 for the HECS-S and 87 for the MDS-R. Almost all nurses were females (n = 88; 95 %). As shown in Table 1, most nurses had worked in paediatrics for more than 5 years (n = 73; 79 %) and over half of the nurses had completed continued education (n = 56; 60 %).

**Table 1.** Years in paediatric healthcare and continued education of the nurses, n = 93.

	n (%)
Years in paediatrics	
<1 year	9 (10)
1–2 years	4 (4)
3–4 years	7 (8)
5–10 years	14 (15)
>10 years	59 (63)
Continued education	
No	37 (40)
Yes, paediatrics	19 (20)
Yes, paediatric oncology	19 (20)
Yes, paediatrics and paediatric oncology	8 (9)
Yes, oncology	4 (4)
Yes, other	4 (4)
Yes, other and paediatric oncology	2 (2)

### Reliability of the instruments

A good internal consistency was confirmed with a Cronbach’s alpha of 0.842 for the Finnish HECS-S and a Cronbach’s alpha of 0.922 for the intensity scale and 0.862 for the frequency scale for the Finnish MDS-R.

### Nurses’ perceptions of the ethical climate

In general, nurses’ perceptions of the ethical climate were positive. The respondent mean for all 18 items, on a scale of 1 to 5, was 4.0 (SD, 0.4). None of the items had a mean score below the midpoint of 3. The highest scored items reflected relationships between co-workers and relations to patients and to parents. Perceptions of working with competent co-workers had the highest mean (4.8; SD, 0.4), as seen in Table 2. The items that reflected perceptions related to hospital policies and conflicts were scored lowest, but with a wider variation between individual nurses. Overall, items assessing relationships to managers were scored lower than items assessing relationships to co-workers.

**Table 2.** The 18 Ethical Climate items, presented in abbreviated forms, in descending order according to means.

Abbreviated items	Mean (SD)
Competent co-workers	4.83 (0.38)
Co-workers listen	4.72 (0.50)
Physicians and nurses trust	4.58 (0.58)
Patients’ wishes	4.49 (0.56)
Parents’ wishes	4.49 (0.56)
Openness asking questions and learning	4.25 (0.67)
I can practise care as it should be	4.16 (0.60)
Hospital’s values shared	4.09 (0.67)
Physicians ask nurses for their opinions	4.08 (0.82)
Ethical problems identified	4.01 (0.78)
Manager I trust	4.00 (0.93)
Physicians and nurses respect each other’s opinions	3.96 (0.71)
Feelings and values taken into account	3.87 (0.73)
Dealing with ethical problems	3.64 (0.90)
Immediate manager helps	3.51 (1.16)
Immediate manager helps my co-workers	3.49 (0.98)
Hospital policies help	3.30 (0.80)
Conflicts openly dealt with	3.24 (0.95)

### Nurses’ perceptions of moral distress

#### Perceived intensities

Overall, the disturbances of the situations described in the MDS-R were scored higher than the frequencies. Nurses assessed almost all scenarios as very disturbing; the respondent mean for intensities was 3.1 (SD, 0.5) on a scale of 0–4. All 26 items had a mean intensity score that exceeded the midpoint of 2.

#### Perceived frequencies

Many nurses perceived several situations described in the MDS-R as uncommon; 12 items out of 26 had a mean frequency score below 1 on a scale of 0–4. The situations that rarely or never occurred involved witnessing unethical behaviour, end-of-life care, hierarchies of decision-making, lack of competence and poor communication. The overall mean score for frequencies was 1.1 (SD, 0.4). The situation involving procedures on resistant school-aged children was the only item with a mean frequency score above the midpoint of 2 (mean, 2.5; SD, 1.1). Other situations, that were scored as moderately frequent, reflected lack of resources: unsafe staffing levels, shortage of time and cost reductions (Table 3).

**Table 3.** The 26 Moral Distress items in descending order ranked by frequency scores.

Abbreviated items	Mean (SD)	
	Frequency	Intensity
Perform painful/unpleasant procedures on school-aged children who resist such treatment	2.51 (1.07)	2.58 (0.95)
Not having time to conduct conversations with patients and families in a way you think they should be carried out	1.98 (1.08)	3.18 (0.79)
Being unable to provide best possible care…pressures from management to reduce costs	1.88 (1.14)	3.03 (1.04)
Work in a staffing situation (number/competence level) that you experience as unsafe	1.83 (1.11)	3.55 (0.61)
Provide care although parents have unrealistic expectations of health care	1.68 (0.81)	2.76 (0.81)
See that…care suffers because of lack of continuity, with many different healthcare providers	1.49 (1.06)	3.03 (0.84)
Not to talk about death with a dying child although you think it is necessary	1.32 (0.96)	3.40 (0.72)
Continue to participate in life-sustaining treatment of a dying child because no one has decided to end that treatment	1.30 (0.99)	2.88 (0.89)
Feel pressured to perform tests and treatments…unnecessary	1.30 (0.93)	2.74 (1.00)
See…professionals give ‘false hope’ to parents	1.29 (0.94)	2.78 (1.06)
Follow family’s wishes…life-sustaining treatment…not in the best interest of the child	1.21 (0.89)	2.66 (0.89)
Follow family’s request not to talk about death…dying child who asks about dying	1.20 (0.94)	3.34 (0.84)
Be expected to care for patients…not feel competent enough to care for	1.20 (0.86)	3.36 (0.89)
See that the quality of patient care suffers because of poor communication within the team	1.00 (0.79)	3.31 (0.75)
Work with nurses…not as competent as…healthcare requires	0.99 (0.81)	3.41 (0.74)
Decide on care/treatment when you are uncertain about what is right	0.87 (0.72)	3.08 (0.84)
Work with a physician…incompetent in providing healthcare	0.80 (0.79)	3.28 (0.91)
Take life-saving actions…only prolong dying	0.76 (0.77)	2.73 (0.85)
Provide inadequate care…not relieve…suffering…physician afraid…will lead to death	0.69 (0.80)	3.68 (0.56)
Give an increased dose of sedatives/opiates…believe…hasten death	0.65 (0.84)	2.44 (1.05)
Avoid reporting…discover that a physician or a nurse has made a medical error	0.62 (0.74)	3.30 (0.88)
Follow physician’s request not to discuss…prognosis with parents	0.49 (0.75)	2.77 (1.09)
Follow the family’s wishes for the child’s care despite…you disagree…afraid of being reported	0.42 (0.68)	3.01 (1.02)
Shut eyes to that parents have not received…information…to give their consent to healthcare…	0.35 (0.53)	3.30 (0.77)
Take no action about an ethical issue…because the involved…professional or management requests…	0.34 (0.59)	3.41 (0.74)
See students perform painful procedures…solely to improve skills	0.32 (0.64)	3.32 (1.04)

### Differences according to work experience in paediatrics and education

Overall, nurses with more than 5 years of work experience in paediatric healthcare assessed situations as significantly (U = 874, p = 0.008) more frequent (mean, 1.2; SD, 0.4) than nurses with less than 5 years of experience (mean, 0.8; SD, 0.4). The perceptions of intensities averaged 3.1 (SD, 0.5) in both groups. Perceptions did not differ significantly between nurses with basic education and nurses with continued education.

### Correlation between the ethical climate and moral distress

There was a moderate, negative correlation between the perceptions of ethical climate and moral distress frequencies (r = −0.435, n = 86, p < 0.001). No significant correlation was found between the perceptions of ethical climate and moral distress intensity scores (r = 0.032, n = 86, p = 0.769). The pattern that shows differences between centres can be seen in Table 4.

**Table 4.** The ethical climate and moral distress intensity and frequency for each centre.

Centre	HECS-S Mean (SD)	MDS-R intensity Mean (SD)	MDS-R frequency Mean (SD)
Centre A	4.35 (0.24)	3.04 (0.71)	0.94 (0.43)
Centre B	4.10 (0.35)	3.15 (0.31)	1.04 (0.30)
Centre C	4.03 (0.38)	3.01 (0.40)	1.05 (0.49)
Centre D	4.00 (0.47)	3.21 (0.49)	1.16 (0.43)
Centre E	3.74 (0.23)	3.04 (0.44)	1.26 (0.32)

HECS-S: Hospital Ethical Climate Survey–Shortened; MDS-R: Moral Distress Scale–Revised.

## Discussion

In this study, we surveyed nurses working in Finnish paediatric oncology care and explored their perceptions of ethical climate and moral distress. Overall, the perceptions of the ethical climate in paediatric oncology centres were positive, and the majority perceived that they most often could practise nursing the way they believed it should be practised. Clinical situations generating moral distress were perceived as quite uncommon, but highly disturbing. An association between ethical climate and frequencies of morally distressing situations was identified.

Nurses in this study shared a positive view on ethical climate. Even in previous studies, perceptions of ethical climate have been mostly positive, according to Koskenvuori et al.^6^ As a part of our current project, ethical climate had already been investigated in paediatric oncology care in Sweden.^14^ Those results suggested positive perceptions in general, but they also revealed that physicians had more positive perceptions than nurses. Compared to nurses in Sweden, Finnish nurses perceived the ethical climate as slightly more positive. The most favourable perceptions reflected relationships to co-workers, and in particular their competence. This probably mirrors the lengthy clinical experience most nurses possessed. Although cross-sectional studies must be interpreted with caution, it could be argued that promoting collegiality and retaining skilled nurses may help to build a positive ethical climate. In any case, medical knowledge and nursing skills need to be kept up to date, and thus, ongoing learning activities are needed to nurture the professional development of nurses.^27^

In accordance with prior findings,^9,14^ nurses perceived that conflicts were not always openly dealt with at their workplaces. This tendency may partly depend on how the expression ‘openly dealt with’ has been interpreted, as some concerns might require privacy. Conflicts involving unprofessional confrontations in front of others have been characterised as destructive.^28^ Even though conflicts were not always openly dealt with, our results suggest that conflicts were more often dealt with than avoided. This is coherent with Johansen and Cadmus^29^ who reported that only one out of four nurses used an avoidant conflict management style.

The hospital policies were not always found helpful in difficult patient care situations. This perception is in line with previous studies.^9,14,30^ One possible explanation to this moderate perception could be that complex healthcare situations often require other resources than policies alone. Facilitated ethics discussions and clinical ethics support services can aid care teams to clarify complex ethical problems.^31^ Winter et al.^32^ found paediatric ethics consultations as rare, but when requested, they most often concerned issues related to non-beneficial treatments. According to Leland et al.,^33^ many paediatric ethics consultations involve provider moral distress. Fortunately, nurses consider formal ethics support as helpful in resolving moral distress.^34^

We found that disagreements on end-of-life care as a root cause of moral distress were not quite as prominent in paediatric oncology units as those reported in critical care settings.^8,21^ Although grief and loss are unavoidable in paediatric oncology nursing,^35^ not all emotionally distressing situations generate moral distress. It is also important to bear in mind that even if paediatric oncology nurses’ experiences of end-of-life care include poor communication, limited truth-telling and cultural obstacles,^36^ moral distress is not inevitable in all end-of-life care. It is possible that qualitative research methods would better capture experiences of moral distress in end-of-life care, and further studies using interview methodology are needed to develop a full picture on the topic. However, morally distressing situations during this vulnerable time should never be overlooked or belittled, as nurses assessed them as highly distressing if or when they occur, and their impact on nurses can be adverse and long-lasting.

The situations our study identified as major sources of moral distress in paediatric oncology nursing reflected performing procedures on school-aged children who resist such treatment, nurses’ lack of time and inadequate staffing levels. Our results describe lack of resources, compared to prior findings,^9,10^ and unfortunately, they mirror the worldwide situation in nursing with heavy workloads and nursing shortages.^37^ Recently, Hopia and Heino-Tolonen^38^ described that busy work shifts caused concerns among paediatric oncology nurses, as a lack of time stopped them from supporting families in a way they felt was needed. Similarly, Newman et al.^39^ reported that failing to provide compassionate, high-quality care escalated moral distress among paediatric oncology nurses. How to balance a healthy work environment with challenging ethical issues and financial cut-backs is a puzzle, but nurturing an ethical climate and collegiality may help to withstand moral distress. Sharing troublesome experiences with peers helps nurses to endure moral distress,^40^ and evidently, communication and collaboration correlate not only with reduced levels of moral distress but also with improved quality of care.^39^

Uncomfortable procedures against children’s will, and the potential infringement on children’s autonomy, presents an ethical challenge in paediatric nursing care.^1^ Physical restraint of small children is perceived to be quite common and distressing.^41^ According to the nurses in our study and earlier findings from Sweden,^15^ even older children resist treatments quite often, but compared to the Swedish results, nurses in Finland did not find these situations quite as disturbing as nurses in Sweden did. This item was added in the Swedish MDS-R,^15^ and the high scores show its relevance in paediatric nursing studies. Although our results do not reveal reasons for resisting and unwillingness among school-aged children, Bray et al.^42^ have shown that children value detailed information about the planed procedure and the individual coping strategies they can use. Thus, it should be considered whether improved dialogue and preparations could prevent some of these situations.

Prior studies have reported conflicting results on moral distress and its relation to nurses’ education and work experience.^43^ These relationships have been studied by many scholars without proof of significant associations,^44,45^ while some have observed that nurses with longer experience report higher levels of moral distress.^46^ Our results indicate a link between years in nursing and frequencies of distressing situations. This could be explained by the rarity of the situations: apparently it will take some time before novice nurses have experienced the situations described in the MDS-R. However, neither nurses’ level of education nor years in paediatric healthcare affected the level of disturbance. Maybe the repetitive exposure to distressing situations over time is not solely disadvantageous. According to Helmers et al.,^34^ it can broaden the understanding of different ways of dealing with ethical dilemmas, leading to a wider perspective on ethical issues that in turn can be helpful in diminishing experiences of moral distress.

Our study supports the proposed relationship between ethical climate and moral distress.^44,47,48^ However, we found this relationship significant only when comparing to moral distress frequencies. This pattern can be clearly noticed by comparing centres: the more positive the ethical climate, the less frequent the experiences of distressing situations. Whether it is an absence of distressing situations that contributes to more positive perceptions of ethical climate, or the other way around, remains uncertain, as the methodological limitations of cross-sectional, correlational studies make it difficult to draw conclusions on causality.

The main limitation of this study is the low response rate. Despite the diligent participation in some centres and the reminders sent by the local coordinators during data collection periods, the overall response rate remained low. Since we did not gather identifying information in the form of nurses’ names or ages, or length of work experience in paediatric oncology, it was not possible to identify the non-responders. Another limitation is the geographic context. Some perceptions are sensitive to social and cultural influences, and therefore, the findings might not be generalizable to other countries.

## Conclusion

Taken together, paediatric oncology nurses share a positive view of the ethical climate and especially of their co-workers’ competence and support. Morally distressing situations described in the MDS-R are, in general, more disturbing than frequent. Lack of time and inadequate staffing levels continue to be among the most frequent, morally distressing situations. These findings highlight the importance of good collegial relationships and suggest that continued efforts are needed to secure manageable workloads for the nurses to reduce moral distress. Finally, this study adds to a growing body of evidence that indicates a negative correlation between ethical climate and moral distress. Since this study is cross-sectional, it is not possible to explain the causality. Therefore, further studies are needed to better understand this association and to provide suggestions on how to best apply this knowledge in paediatric oncology nursing.

## References

[1] Bartholdson C Lutzen K Blomgren K, et al. Experiences of ethical issues when caring for children with cancer. Cancer Nurs 2015; 38(2): 125–132. 24945260 10.1097/NCC.0000000000000130

[2] Olson L. Ethical climate in health care organizations. Int Nurs Rev 1995; 42(3): 85–90. 7649724

[3] Victor B Cullen J. A theory and measure of ethical climate in organizations. In: Frederick W Preston L (eds) Research in corporate social performance and policy. Greenwich, CT: Jai Press, 1987, pp. 51–71.

[4] Olson LL. Hospital nurses’ perceptions of the ethical climate of their work setting. Image J Nurs Sch 1998; 30(4): 345–349. 9866295 10.1111/j.1547-5069.1998.tb01331.x

[5] Bartholdson C Sandeberg MA Lutzen K, et al. Healthcare professionals’ perceptions of the ethical climate in paediatric cancer care. Nurs Ethics 2016; 23(8): 877–888. 26116632 10.1177/0969733015587778

[6] Koskenvuori J Numminen O Suhonen R. Ethical climate in nursing environment: a scoping review. Nurs Ethics 2019; 26(2): 327–345. 28659068 10.1177/0969733017712081

[7] Jameton A. A reflection on moral distress in nursing together with a current application of the concept. J Bioeth Inq 2013; 10: 297–308. 24048753 10.1007/s11673-013-9466-3

[8] Sannino P Gianni ML Carini M, et al. Moral distress in the pediatric intensive care unit: an Italian study. Front Pediatr 2019; 7: 338. 10.3389/fped.2019.00338 PMC6700377 31456996

[9] Sauerland J Marotta K Peinemann MA, et al. Assessing and addressing moral distress and ethical climate part II. Dimens Crit Care Nurs 2015; 34(1): 33–46. 25470266 10.1097/DCC.0000000000000083

[10] Pergert P Bartholdson C Blomgren K, et al. Moral distress in paediatric oncology: Contributing factors and group differences. Nurs Ethics 2019; 26(7–8): 2351–2363. 30411660 10.1177/0969733018809806

[11] Lazzarin M Biondi A Di Mauro S. Moral distress in nurses in oncology and haematology units. Nurs Ethics 2012; 19(2): 183–195. 22457383 10.1177/0969733011416840

[12] Oh Y Gastmans C. Moral distress experienced by nurses: a quantitative literature review. Nurs Ethics 2015; 22(1): 15–31. 24091351 10.1177/0969733013502803

[13] Karakachian A Colbert A. Nurses’ moral distress, burnout, and intentions to leave: an integrative review. J Forensic Nurs 2019; 15(3): 133–142. 31436681 10.1097/JFN.0000000000000249

[14] Pergert P Bartholdson C af Sandeberg M. The ethical climate in paediatric oncology- a national cross-sectional survey of health-care personnel. Psychooncology 2019; 28(4): 735–741. 30695112 10.1002/pon.5009 PMC6594059

[15] af Sandeberg M Bartholdson C Pergert P. Important situations that capture moral distress in paediatric oncology. BMC Med Ethics 2020; 21, 6. 31931787 10.1186/s12910-020-0447-x PMC6958740

[16] Hamric AB Blackhall LJ. Nurse-physician perspectives on the care of dying patients in intensive care units: collaboration, moral distress, and ethical climate. Crit Care Med 2007; 35(2): 422–429. 17205001 10.1097/01.CCM.0000254722.50608.2D

[17] Whitehead P Herbertson R Hamric A, et al. Moral distress among healthcare professionals: report of an institution-wide survey. J Nurs Scholarsh 2015; 47(2): 117–125. 25440758 10.1111/jnu.12115

[18] Pergert P Bartholdson C Wenemark M, et al. Translating and culturally adapting the shortened version of the Hospital Ethical Climate Survey (HECS-S) – retaining or modifying validated instruments. BMC Med Ethics 2018; 19, 35. 29747639 10.1186/s12910-018-0274-5 PMC5946534

[19] Corley M Elswick R Gorman M, et al. Development and evaluation of a moral distress scale. J Adv Nurs 2001; 33(2): 250–256. 11168709 10.1046/j.1365-2648.2001.01658.x

[20] Hamric AB Borchers CT Epstein EG. Development and testing of an instrument to measure moral distress in healthcare professionals. AJOB Prim Res 2012; 3: 1–9.26137345

[21] Larson C Dryden-Palmer K Gibbons C, et al. Moral distress in PICU and neonatal ICU practitioners: a cross-sectional evaluation. Pediatr Crit Care Med 2017; 18(8): e318–e326. 28598947 10.1097/PCC.0000000000001219

[22] af Sandeberg M Wenemark M Bartholdson C, et al. To change or not to change – translating and culturally adapting the paediatric version of the Moral Distress Scale-Revised (MDS-R). BMC Med Ethics 2017; 18, 14. 28219363 10.1186/s12910-017-0176-y PMC5319143

[23] Daniel M Miller A Wilbur J. Multiple instrument translation for use with South Asian Indian immigrants. Res Nurs Health 2011; 34(5): 419–432. 21818758 10.1002/nur.20450 PMC3171609

[24] Willis GB.Cognitive interviewing: a ‘how to’ guide. In: Short course presented at the meeting of the American Statistical Association, 1999, https://www.hkr.se/contentassets/9ed7b1b3997e4bf4baa8d4eceed5cd87/gordonwillis.pdf

[25] Suhonen R Stolt M Katajisto J, et al. Validation of the hospital ethical climate survey for older people care. Nurs Ethics 2015; 22(5): 517–532. 25316460 10.1177/0969733014549878

[26] World Medical Association. WMA declaration of Helsinki – ethical principles for medical research involving human subjects, https://www.wma.net/policies-post/wma-declaration-of-helsinki-ethical-principles-for-medical-research-involving-human-subjects/ (accessed Oct 27 2020). 19886379

[27] Hopia H Miettinen S Miettinen M, et al. The voice of paediatric oncology nurses: a longitudinal diary study of professional development. Eur J Oncol Nurs 2019; 42: 28–35. 31446261 10.1016/j.ejon.2019.07.009

[28] Kim WS Nicotera AM McNulty J. Nurses’ perceptions of conflict as constructive or destructive. J Adv Nurs 2015; 71: 2073–2083. 25892228 10.1111/jan.12672

[29] Johansen ML Cadmus E. Conflict management style, supportive work environments and the experience of work stress in emergency nurses. J Nurs Manag 2016; 24(2): 211–218. 25846993 10.1111/jonm.12302

[30] Constantina C Papastavrou E Charalambous A. Cancer nurses’ perceptions of ethical climate in Greece and Cyprus. Nurs Ethics 2018; 26: 1805–1821. 29734886 10.1177/0969733018769358

[31] Rasoal D Skovdahl K Gifford M, et al. Clinical ethics support for healthcare personnel: an integrative literature review. HEC Forum 2017; 29(4): 313–346. 28600658 10.1007/s10730-017-9325-4 PMC5688194

[32] Winter MC Friedman DN McCabe MS, et al. Content review of pediatric ethics consultations at a cancer center. Pediatr Blood Cancer 2019; 66(5): e27617. 30666797 10.1002/pbc.27617 PMC6777958

[33] Leland BD Wocial LD Drury K, et al. Development and retrospective review of a pediatric ethics consultation service at a large academic center. HEC Forum 2020; 32(3): 269–281. 32180057 10.1007/s10730-020-09397-6

[34] Helmers A Palmer KD Greenberg RA. Moral distress: developing strategies from experience. Nurs Ethics 2020; 27(4): 1147–1156. 32238031 10.1177/0969733020906593

[35] Boyle D Bush NJ. Reflections on the emotional hazards of pediatric oncology nursing: four decades of perspectives and potential. J Pediatr Nurs 2018; 40: 63–73. 29776481 10.1016/j.pedn.2018.03.007

[36] Montgomery KE Sawin KJ Hendricks-Ferguson V. Communication during palliative care and end of life: perceptions of experienced pediatric oncology nurses. Cancer Nurs 2017; 40(2): E47–E57. 27044058 10.1097/NCC.0000000000000363

[37] World Health Organization. State of the world’s nursing 2020: investing in education, jobs and leadership. https://www.who.int/publications/i/item/9789240003279 (accessed Oct 21 2020).

[38] Hopia H Heino-Tolonen T. Families in paediatric oncology nursing: critical incidents from the nurses’ perspective. J Pediatr Nurs 2019; 44: e28–e35. 30528181 10.1016/j.pedn.2018.10.013

[39] Newman AR Callahan MF Lerret SM, et al. Pediatric oncology nurses’ experiences with prognosis-related communication. Oncol Nurs Forum 2018; 45: 327–337. 29683123 10.1188/18.ONF.327-337

[40] Passos Dos Santos R Tatsch Neves E Carnevale F. The moral experiences of pediatric nurses in Brazil: engagement and relationships. Nurs Ethics 2019; 26(5): 1566–1578. 29495934 10.1177/0969733017753744

[41] Lombart B De Stefano C Dupont D, et al. Caregivers blinded by the care: a qualitative study of physical restraint in pediatric care. Nurs Ethics 2020; 27(1): 230–246. 30975025 10.1177/0969733019833128

[42] Bray L Appleton V Sharpe A. The information needs of children having clinical procedures in hospital: will it hurt? Will I feel scared? What can I do to stay calm? Child Care Health Dev 2019; 45(5): 737–743. 31163093 10.1111/cch.12692 PMC6851850

[43] McAndrew NS Leske J Schroeter K. Moral distress in critical care nursing: the state of the science. Nurs Ethics 2018; 25(5): 552–570. 27660185 10.1177/0969733016664975

[44] de Boer JC van Rosmalen J Bakker AB, et al. Appropriateness of care and moral distress among neonatal intensive care unit staff: repeated measurements. Nurs Crit Care 2016; 21(3): e19–e27. 26380963 10.1111/nicc.12206

[45] Larson CP Dryden-Palmer KD Gibbons C, et al. Moral distress in PICU and neonatal ICU practitioners: a cross-sectional evaluation. Pediatr Crit Care Med 2017; 18(8): e318–e326. 28598947 10.1097/PCC.0000000000001219

[46] Marturano ET Hermann RM Giordano NA, et al. Moral distress: identification among inpatient oncology nurses in an academic health system. Clin J Oncol Nurs 2020; 24: 500–508. 32945796 10.1188/20.CJON.500-508

[47] Altaker KW Howie-Esquivel J Cataldo JK. Relationships among palliative care, ethical climate, empowerment, and moral distress in intensive care unit nurses. Am J Crit Care 2018; 27(4): 295–302. 29961665 10.4037/ajcc2018252

[48] Epstein EG Whitehead PB Prompahakul C, et al. Enhancing understanding of moral distress: the measure of moral distress for health care professionals. AJOB Empir Bioeth 2019; 10: 113–124. 31002584 10.1080/23294515.2019.1586008

